# Supplementary material for: Conditional chemoconnectomics (cCCTomics) as a strategy for efficient and conditional targeting of chemical transmission
Source: eLife. 2024 Apr 30;12:RP91927. doi: 10.7554/eLife.91927 (PMC11060718; doi:10.7554/eLife.91927)
Supplement: Figure 3—source data 3. [file elife-91927-fig3-data3.zip › Figure 3 source data3 part 1.zip!/Figure 3-Source data 3-part1/1063-13-CRISPResso_Report_8cz_fsdr.zip!/CRISPResso_on_1063_13/1a.Read_barplot.pdf]

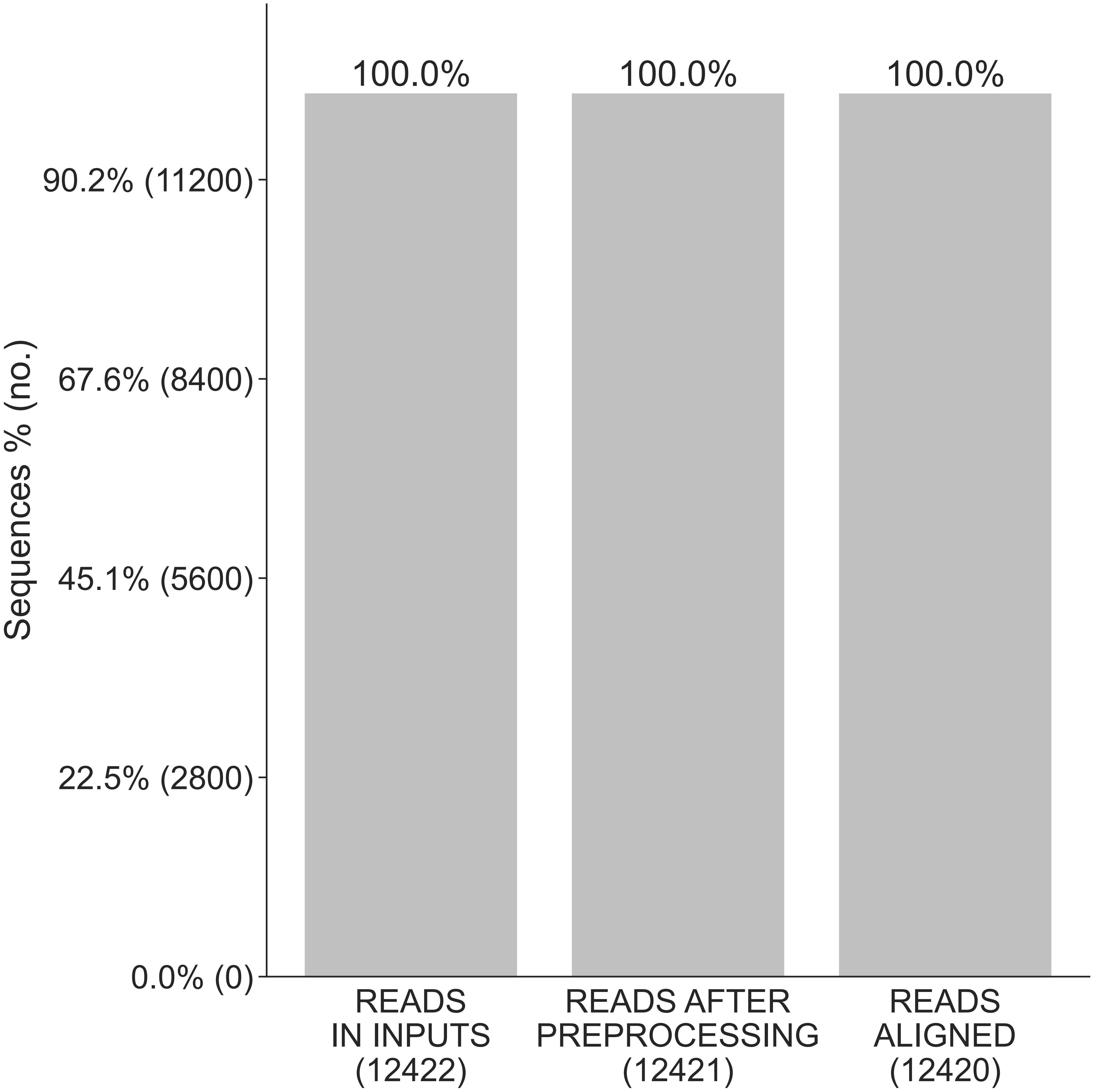
100.0%
100.0%
100.0%
Sequences % (no.)
90.2% (11200)
67.6% (8400)
45.1% (5600)
22.5% (2800)
0.0% (0)
READS IN INPUTS (12422)
READS AFTER PREPROCESSING (12421)
READS ALIGNED (12420)
